# High dynamic range capillary electrophoresis method for sensitive detection of low-frequency driver mutations

**DOI:** 10.1038/s41598-025-01884-5

**Published:** 2025-07-01

**Authors:** Nobue Tamamura, Yoshihiko Hagiwara, Hirokazu Kato, Yusuke Ono, Kenji Takahashi, Kazuya Koyama, Hiroki Sato, Tetsuhiro Okada, Hidemasa Kawabata, Yu Ohtaki, Chiho Maeda, Miyuki Mori, Shin-ichi Chiba, Mishie Tanino, Kenzui Taniue, Takashi Anazawa, Ryoji Inaba, Yusuke Mizukami

**Affiliations:** 1 Department of Advanced Genomic Community Healthcare, Asahikawa Medical University, Asahikawa, Hokkaido 078-8510 Japan; 2 Division of Gastroenterology, Department of Medicine, Asahikawa Medical University, 2-1 Midorigaoka Higashi, Asahikawa, Hokkaido 078-8510 Japan; 3 Naka Diagnostic Products Division, Bio Systems Design 1st Department, Hitachi High-Tech Corporation, Toranomon Hills Business Tower, 1-17-1 Toranomon, Minato-ku, Tokyo, 105-6409 Japan; 4 Institute of Biomedical Research, Sapporo-Higashi Tokushukai Hospital, Sapporo, Hokkaido 065-0033 Japan; 5 Center for Advanced Research and Education, Asahikawa Medical University, Asahikawa, Hokkaido 078-8510 Japan; 6 Department of Diagnostic Pathology, Asahikawa Medical University Hospital, Asahikawa, Hokkaido 078-8510 Japan; 7 Isotope Science Center, The University of Tokyo, Tokyo, 113-0032 Japan; 8 Hitachi, Ltd., Tokyo, 185-8601 Japan

**Keywords:** HiDy, Capillary gel electrophoresis, Dynamic range, Low-frequency mutation, KRAS, Fragment analysis, Gastroenterology, Biomarkers, Diagnostic markers

## Abstract

Cancer genomics aims to personalize treatments by identifying genetic abnormalities in cancer cells. However, current analytical techniques face limitations in simplicity and cost-effectiveness. To address these issues, we developed an enhanced capillary gel electrophoresis (CE) sequencer using a fluorescence-acquisition technique called “HiDy” (High Dynamic range) (HiDy-CE). The HiDy-CE reduces the hardware binning region size and increases the number of regions on a charge-coupled device image sensor, expanding the dynamic range and reducing saturation risk. By applying the multi-base primer extension method to the HiDy-CE with control DNA containing known mutations, we detected variant allele frequencies (VAFs) as low as 0.5% for major *KRAS* hotspot mutation at codon 12 and 13. With 10 ng of DNA from small tissues obtained via fine-needle biopsy from patients with suspected pancreaticoduodenal tumors, HiDy-CE produced equivalent VAFs in *KRAS* compared with targeted amplicon sequencing. This demonstrated the world’s first capability of detecting mutations below 1% on CE using pathological specimens, leveraging its wide dynamic range. With only 2 ng of input DNA, the HiDy-CE provided results highly concordant with digital PCR with minimal non-specific noise. These findings underscore the HiDy-CE’s potential for sensitive detection of oncogenes such as *KRAS*, facilitating pre-testing before comprehensive genome profiling.

## Introduction

Molecular cancer diagnosis is essential for developing effective treatment strategies and accurate prognostic predictions, fueled by rapid advancements in diagnostic techniques that enable precision medicine. While imaging modalities and pathological assessment provide valuable spacial and structural information about tumors, integrating biomarkers enables more precise cancer diagnosis based on tumor biology and subtyping^1^. Although comprehensive genomic profiling has limited widespread benefits in selecting candidates for specific molecular-targeted therapies, using a multimodal approach with careful selection of patient ranges during pre-testing may offer valuable insight^2^. Consequently, addressing the challenge of establishing accurate, highly sensitive mutation quantification, and cost-effective molecular screening for early diagnosis remains a priority in cancer research and clinical practice^3^.

Genetic mutations, particularly in oncogenes such as RAS, play a crucial role in tumor initiation and progression. For example, *KRAS* mutations are found in over 90% of pancreatic ductal adenocarcinoma (PDA) cases, representing a characteristic genetic alteration^4^. These mutations often occur early during tumorigenesis, typically preceding invasive stages. Mutations in other RAS genes, such as *NRAS* and *HRAS*, are also prevalent in various cancers. Each mutation type exhibits distinct frequencies and hotspot locations, affecting tumor progression differently across cancer types^5^. The dosage of these mutations significantly impact tumor progression. Alterations in *KRAS* mutation-specific allelic imbalance can enhance tumor aggressiveness by promoting invasion and metastatic capabilities^6^. Therefore, the presence and quantification of these mutations, including those in *KRAS* oncogenes, are essential for accurate tumor diagnosis and understanding their mechanisms of development.

Various techniques have been developed for detecting oncogenic mutations. These include Sanger sequencing of PCR products^7^, PCR-restriction fragment length polymorphism^8^, high-resolution melting analysis coupled with amplification-refractory mutation system (Scorpion assay)^9^, performed using real-time PCR. Additionally the PASEA assay, which enhances target nucleic acid sequences by degrading a large amount of background nucleic acid sequences via CRISPR/Cas9^10^, and the cationic conjugated polymer-based FRET fingerprint spectrum method, which utilizes fluorescence resonance energy transfer (FRET)^11^, are widely employed in research laboratories for identifying single nucleotide variants (SNVs)^12^. Furthermore, the primer extension-based method, SNaPshot, initially described for detecting *KRAS* codons 12 and 13 mutations^13^, enables the simultaneous analysis of up to 50 biallelic SNVs in a single reaction. This method has been extended in multiplex SNaPshot assays to detect mutations in several oncogenes from formalin-fixed and paraffin-embedded (FFPE) samples^14^. However, quantification remains challenging with these methods; detection limits are approximately 10–20% for Sanger sequencing and 1–5% for SNaPshot^7^.

Another method, such as targeted amplicon sequencing, is increasingly becoming the standard for mutation profiling. While molecular barcode methods excel at detecting rare mutations or mutations with low frequencies, they require substantial steps and incur significant costs, complicating data analysis, especially when dealing with large datasets or diverse samples^15^. Although analysis by digital PCR (dPCR) offers high sensitivity and specificity for detecting specific gene mutations, its ability to analyze multiple gene loci simultaneously remains limited^16,17^.

We aimed to enhance the capability of capillary gel electrophoresis (CE) for detecting mutations present at levels below 1%, while leveraging the cost-effectiveness and rapid turnaround time (TAT) inherent to CE sequencing technology. Previous studies^18,19^ achieved High Dynamic Range Capillary Electrophoresis (HiDy-CE) using ultra-small spectrometers. However, integrating such such an ultra-small spectrometer into a conventional-CE sequencer is challenging. Therefore, in this study, we developed a straightforward technique to upgrade a conventional-CE sequencer to HiDy-CE. HiDy-CE is implemented by modifying the operation of the charge-coupled device (CCD) image sensor used for fluorescence detection, without requiring alterations to the existing hardware configuration of a conventional-CE sequencer^20^. This modification raises the fluorescence signal saturation threshold, thereby expanding the dynamic range. We validated the HiDy-CE’s efficacy in detecting low-frequency mutations in *KRAS* genes, which are commonly implicated in tumorigenesis of the pancreas and other organs, using minute tissue specimens obtained from endoscopic ultrasound-guided fine-needle biopsy (EUS-FNB).

## Results

### Expanded dynamic range enables low-frequency mutation detection

**Fig. 1:**
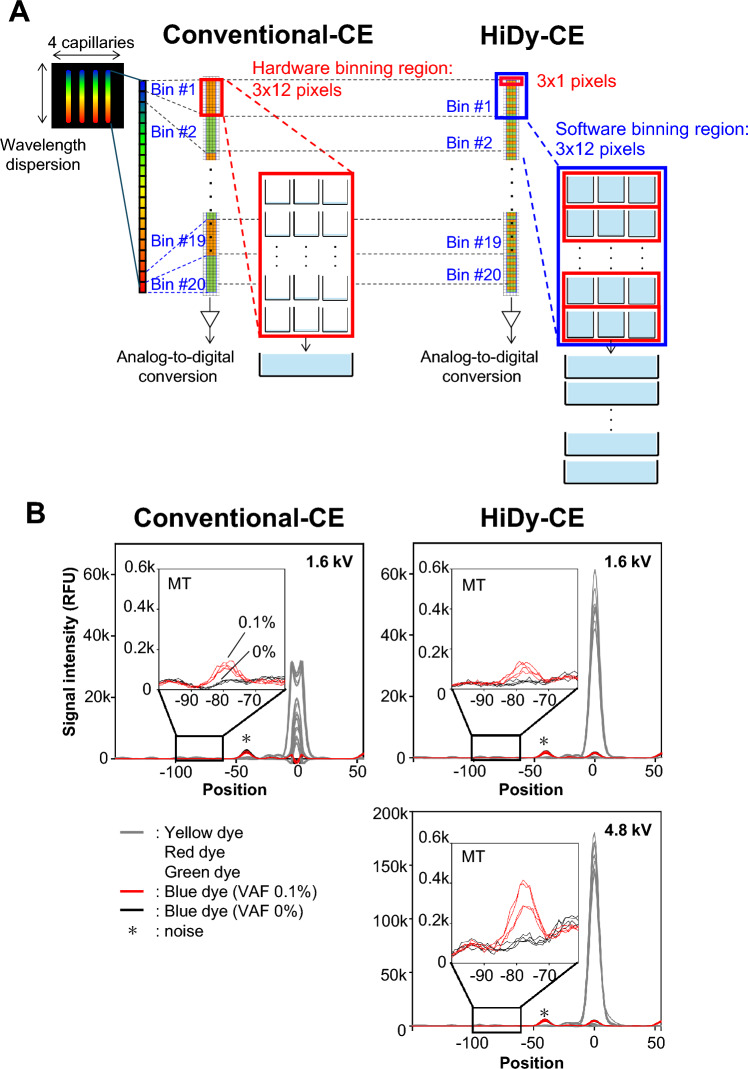
Comparison of the Conventional-CE sequencer and the HiDy-CE sequencer. (**A**) Schematic comparison of binning methods in conventional-CE and HiDy-CE. On each of wavelength-dispersion images of fluorescences from four capillaries, 20 hardware-binning regions of 3 × 12 pixels are set for conventional-CE. Meanwhile, 240 hardware-binning regions of 3 × 1 pixels and 20 software-binning regions of 3 × 12 pixels are set for HiDy-CE. Each software-binning region for HiDy-CE can measure 12 times as much fluorescence as each hardware-binning region for conventional-CE. (**B**) Electropherograms overlay of *KRAS* G12R mutation (MT) analysis using commercially available DNA, prepared with STA, showing MT abundance ratios of 0 and 0.1%. Figures depict four replicate measurements per condition, with peak aligned for clarity. Upper-left panels shows result under standard protocol conditions (1.6-kV injection voltage) for conventional-CE (left) and HiDy-CE (right), demonstrating saturation of wild-type peaks. Lower-left panel illustrates HiDy-CE results with increased injection voltage (4.8 kV) to enhance detection of mutant peak.

We modified a conventional-CE sequencer (compact CE sequencer DS3000, Hitachi High-Tech, Tokyo, Japan) to enhance low-frequency mutation, creating HiDy-CE (Fig. 1A). In a conventional-CE sequencer, fluorescence signals from four capillaries undergo wavelength dispersion and are detected in 3 × 240-pixel region on the CCD image sensor. This 3 × 240-pixel region is divided into 20 hardware-binning regions of 3 × 12 pixels, where analog-to-digital conversion is performed. In contrast, in HiDy-CE, the 3 × 240-pixel region is divided into 240 hardware-binning regions of 3 × 1 pixels. These 240 hardware-binning regions are then grouped in increments of 12, forming a total of 20 software-binning regions of 3 × 12 pixels on the computer. This modification was made possible by faster data acquisition. Since the upper fluorescence detection limit for each hardware-binning region remains the same in the both CE sequencers, the fluorescence detection capacity of each software-binning region in HiDy-CE is 12 times higher than that of an individual hardware-binning region in a conventional CE sequencer. However, software binning slightly increases noise levels. Despite this, the dynamic range of HiDy-CE is 8.09 times greater than that of the conventional CE sequencer.

We compared the two sequencers using control DNA to assess the impact of this enhancement using TrimGen’s Shifted Termination Assay (STA) with control DNA. A *KRAS* G12R mutant peak with variant allele frequency (VAF) of 0.1% was detected in both CE sequencers at an injection volage of 1.6 kV (Fig. 1B). The conventional-CE produced saturated wild-type peaks, preventing precise calculation of VAF (Fig. 1B, upper-left panel). In contrast, the HiDy-CE demonstrated unsaturated wild-type peaks under the same conditions, taking advantage of its broader dynamic range (Fig. 1B, upper-right panel).

Adjusting the injection voltage on the conventional-CE to 0.5 kV prevented wild-type peak saturation but did not enable accurate quantification of low-frequency mutations (Suppl Fig. 1A). Increasing the injection voltage on the HiDy-CE to 4.8 kV expanded the dynamic range, preventing wild-type peak saturation and enabling quantification of *KRAS* mutations with VAFs as low as 0.1% (Fig. 1B, lower-right panel).

### HiDy-CE detects mutations below 1% frequency with control gDNA

**Fig. 2:**
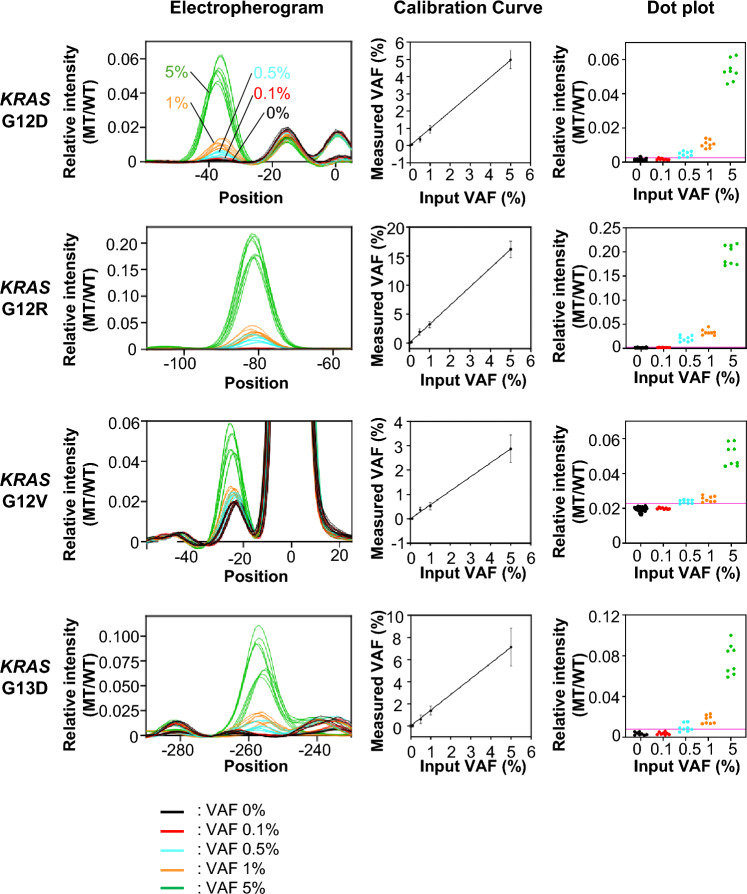
Detection limits and calibration curves for *KRAS* MTs on HiDy-CE using control sample. Left panels: overlay of electropherograms from *KRAS* MTs G12D, G12R, G12V, and G13D analyzed with HiDy-CE at various VAFs (0, 0.1, 0.5, 1, and 5%). Each condition was replicated eight times, with peak position aligned and signal intensity normalized so that maximum intensity of wild-type (WT) peak equals 1. Middle panels: Calibration curves for each MT type, displaying standard deviations (SDs). X-axis represents prepared VAF, while Y-axis shows measured VAF calculated (details are shown in Suppl Fig. 1). Right panels: dot plots of relative intensity (MT/WT) for each condition. Thresholds are set as mean value plus three SDs from baseline (0% input VAF), represented with magenta line. Detection limits for *KRAS* G12D, G12R, G12V, and G13D are indicated in percentages.

To validate the HiDy-CE’s ability to detect low-frequency mutation, we used commercially available genomic DNA (gDNA) containing *KRAS* mutations (G12D, G12R, G12V, and G13D). Serial dilutions with wild-type gDNA (ranging from 0 to 5%) were analyzed using the HiDy-CE. The measured VAFs, i. e., the detected variant allele frequencies, were calculated from the ratio of mutant and wild-type peak-signal intensities (Suppl Fig. 1B). The detection limits for *KRAS* mutations were determined to be 0.5% for G12D and G12R and 1% for G12V and G13D (Fig. 2, right panels). This indicates that the HiDy-CE reliably detects mutations at frequencies below 1% using commercially available DNA.

### Comparison with conventional-CE using pathological specimens

**Fig. 3:**
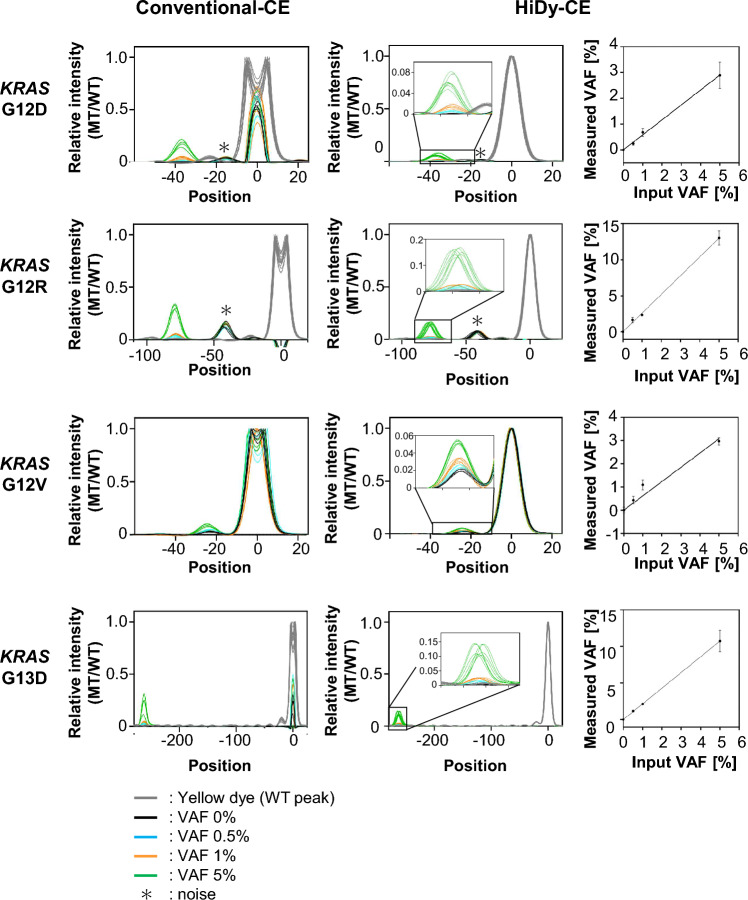
Comparison of Conventional-CE and HiDy-CE for *KRAS* MT using pathological specimens. Left panels: Electropherograms analyzed using conventional-CE for *KRAS* MTs G12D, G12R, G12V, and G13D at VAFs of 0, 0.5, 1, and 5%. Four replicate measurements were conducted for each condition, with peaks aligned at WT peak top. Signal intensities were normalized so maximum intensity of WT peak is 1. WT peaks display saturated due to narrow dynamic range. Middle panels: Electropherograms using HiDy-CE under same conditions as above. Eight replicates were conducted, with peak alignments and intensity normalized like left panels. Wider dynamic range of HiDy-CE prevents WT peak saturation, enabling accurate VAF calculation. Right panels: Calibration curves for each MT type with HiDy-CE, showing SDs. X-axis represents prepared VAF, while Y-axis shows calculated VAF based on protocol illustrated in Suppl Fig. 1. Baseline VAF (0% input) is used as reference point.

We further assessed the mutation detection capability of the HiDy-CE compared with the conventional-CE using patients’ samples from surgically resected tumor and normal pairs of FFPE specimens (Fig. 3). With the conventional-CE, mutant signals were observed but were accompanied by saturated wild-type peaks, complicating precise VAF calculation (Fig. 3, left panels). Reducing the sample input volume did not alleviate wild-type peak saturation, and the electropherogram of the mutant peak was distorted, making precise VAF calculation unfeasible (Suppl Fig. 1C).

The HiDy-CE, with its wider dynamic range, prevented wild-type peak saturation, enabling precise VAF calculation (Fig. 3, right panels). Detection limits for *KRAS* mutations, based on thresholds calculated from DNA derived solely from normal tissues (VAF at 0%), were 0.5% for G12D, G12R, and G13D and 1% for G12V (Suppl Fig. 2, left panels). When thresholds were calculated including the other three non-target variants, detection limits were adjusted to 1% for G12D and G12V and 5% for G12R (Suppl Fig. 2, right panels). These results underscore the HiDy-CE’s capability to detect and quantify mutations in clinical samples at frequencies as low as 1%.

### HiDy-CE precision with EUS-FNB samples

**Fig. 4:**
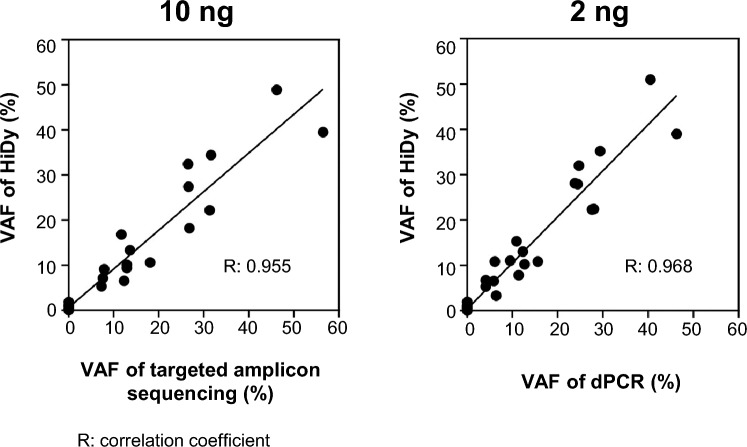
Correlation analysis between HiDy-CE and other molecular techniques. Left panel: Correlation between HiDy-CE and targeted amplicon sequencing for DNA samples quantified at 10 ng. Correlation coefficient is 0.955, indicating strong correlation. Right panel: correlation between HiDy-CE and dPCR for DNA samples quantified at 2 ng. Correlation coefficient is 0.968, reflecting high reliability. Black line in both panels represents best fit line for data points.

We next assessed the HiDy-CE’s effectiveness in detecting mutations from small tissue specimens obtained via EUS-FNB (Table 1). DNA was extracted from a total of 34 specimens, and given the small size of some specimens, the yield was consistent with the tissue amount estimated from Hematoxylin–Eosin (H–E) staining images (Suppl Fig. 3). Twenty-four of the samples yielded sufficient DNA (≥ 10 ng) for STA analysis. Duplicate measurements on the HiDy-CE confirmed all mutations were detected by targeted amplicon sequencing, showing strong agreement between the two methods (Table 1). A high correlation (correlation coefficient of 0.955) was observed between the VAFs obtained from the HiDy-CE and targeted amplicon sequencing (Fig. 4, left panel).

**Table 1:** Mutation detection capability on HiDy-CE using FNB samples.

Patient	Pathologic diagnosis by FNB	DNA yield Qubit (ng)	10 ng				2 ng			
			HiDy		Targeted amplicon sequencing		HiDy		digital PCR	
			*KRAS* mutation	VAF (%)	*KRAS* mutation	VAF (%)	*KRAS* mutation	VAF (%)	*KRAS* mutation	VAF (%)
1	Adenocarcinoma	1122	G12D	9.4	G12D	12.9	G12D	7.8	G12D	11.4
2	Adenocarcinoma	762	G12V*	27.4	G12V	26.6	G12V*	27.9	G12V	24.4
3	Adenocarcinoma	2388	G12R*	13.3	G12R	13.6	G12R	13.0	G12R	12.3
4	Atypical glands (dysplasia)	21	N/A	–	N/A	–	ND		ND	
5	Adenocarcinoma	466	G12D	9.8	G12D	18.1	G12D	10.8	G12D	15.6
6	Adenocarcinoma	535	G12V*	48.9	G12V	46.2	G12V*	51.0	G12V	40.5
7	Adenocarcinoma	840	G12D	10.1	G12D	12.9	G12D	10.2	G12D	12.7
8	Adenocarcinoma	2940	G12V	5.3	G12V	7.3	G12V	6.7	G12V	4.1
9	Chronic pancreatitis	354	ND		ND		ND		ND	
10	Duodenum mucosa	27	N/A	–	N/A	–	ND		ND	
11	Adenocarcinoma	226	G12D	39.5	G12D	56.5	G12D*	39.0	G12D	46.3
12	Adenocarcinoma	1638	ND		ND		ND		ND	
13	Adenocarcinoma	654	G12D	22.2	G12D	31.3	G12D	22.4	G12D	28.0
14	Adenocarcinoma	451	G12V	7.1	G12V	7.6	G12V	6.5	G12V	5.9
15	B cell lymphoma	882	ND		ND		ND		ND	
16	GIST	346	ND		ND		ND		ND	
17	GIST	179	*	*	ND		*	*	ND	
18	Adenocarcinoma	139	G12V*	27.0	G12V	26.8	G12V*	28.1	G12V	23.8
19	GIST	298	ND		ND		ND		ND	
20	Degeneration tissue	126	ND		ND		ND		ND	
21	Adenocarcinoma	74	G12V*	34.4	G12V	31.6	G12V*	35.2	G12V	29.4
			G12R	9.1	G12R	7.9	G12R	10.8	G12R	6.1
22	Adenocarcinoma	798	ND	–	ND		ND		ND	
23	IOPN (high-grade dysplasia)	1320	ND		ND		ND		ND	
24	Adenocarcinoma	210	G12V*	32.4	G12V	26.5	G12V*	32.0	G12V	24.7
			G12R	16.8	G12R	11.7	G12R*	15.3	G12R	10.9
25	Atypical glands (dysplasia)	164	G12R*	12.1	G12R	12.3	G12R	11.0	G12R	9.5
26	Adenocarcinoma	85	G12D	20.5	N/A	–	G12D	22.3	G12D	27.6
27	No evidence of malignancy	22	N/A	–	N/A	–	ND		ND	
28	Insufficient material	25	N/A	–	N/A	–	G12D	5.3	G12D	4.1
29	Insufficient material	39	N/A	–	N/A	–	G12D	3.3	G12D	6.4
30	Insufficient material	36	N/A	–	N/A	–	ND		ND	
31	No evidence of malignancy	432	N/A	–	N/A	–	ND		ND	
32	No evidence of malignancy	179	N/A	–	N/A	–	ND		ND	
33	No evidence of malignancy	726	N/A	–	N/A	–	ND		ND	
34	No evidence of malignancy	180	N/A	–	N/A	–	*	*	ND	

*VAF* Variant Allele Frequency, *N/A* not analyzed, *ND* not detected; *, including mutation detected at HiDy-CE but not detected at amplicon-targeted sequencing or digital PCR; GIST, gastrointestinal stromal tumor; IOPN, intraductal oncocytic –papillary neoplasm.

We also tested the HiDy-CE’s capability to quantify mutations with minimal DNA input (2 ng), comparing the results with those from dPCR. Despite the lower DNA amount, the HiDy-CE showed consistent results, with a high correlation coefficient (0.968) compared with dPCR (Fig. 4, right panel). Notably, mutations were detectable on both the HiDy-CE and dPCR, even in samples with inconclusive pathological diagnosis (Table 1). While some non-specific mutations were detected, the HiDy-CE accurately identified all mutations, demonstrating efficacy even with minimal DNA input (Suppl Table 1).

### Low-frequency mutation detection in diluted samples

**Fig. 5:**
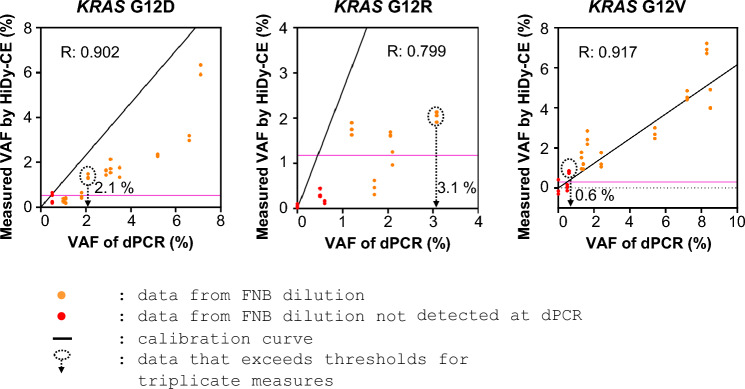
Detection limits for *KRAS* MTs in diluted EUS-FNB samples. Diagrams examining detection thresholds for *KRAS* G12D, G12R, and G12V in EUS-FNB-derived DNA diluted with commercially available DNA. Triplicate measurements were conducted for each dilution series, plotting VAF of dPCR against that of HiDy-CE on X- and Y-axes, respectively. In dPCR measurements, if the number of MT droplets exceeds three, it is judged as harboring mutation. If the number of MT droplet is three or less, the VAF of dPCR is plotted as reference value. Detection limits were established where triplicate data consistently exceeded thresholds from clinical samples (see Suppl Fig. 2, right panels), determining detection limits at 2.1% for *KRAS* G12D, 3.1% for G12R, and 0.6% for G12V when diluting EUS-FNB sample with commercially available DNA. Thresholds of VAF were calculated from thresholds those of MT/WT (see Suppl Fig. 2, right panels) and calibration curves (see Fig. 3, right panels). Calibration curves are established from clinical samples (see Fig. 3, right panels).

Assessing the HiDy-CE’s capability to detect low-frequency mutations, EUS-FNB samples diluted to less than VAF 5% with wild-type gDNA were analyzed using the HiDy-CE and dPCR. Triplicate measurements on the HiDy-CE revealed detection limits for *KRAS* mutations as 2.1% for G12D, 3.1% for G12R, and 0.6% for G12V (Fig. 5), as calculated from data measured using dPCR, and 0.56% for G12D, 2.1% for G12R, and 0.81% for G12V (Suppl Fig. 4), as calculated from theoretical estimation obtained through targeted amplicon sequencing before dilution. Notably, no non-specific mutations were detected in the diluted DNA. A strong correlation (correlation coefficients ranging from 0.799 to 0.917) (Fig. 5) was observed between the VAFs obtained from the HiDy-CE and the input VAFs, highlighting the reliability and accuracy of the HiDy-CE in detecting and quantifying low-frequency mutations compared with established modalities such as dPCR.

## Discussion

We introduced the HiDy-CE, a novel sequencing technology capable of detecting lower-frequency genetic mutations. We validated its efficacy using pathological specimens and demonstrated its capability to detect mutations at frequencies less than 1%. This unique wide dynamic range sequencer will pave the way for achieving multiplex lower-frequency mutation detection with CE, which would not be possible with the conventional-CE.

Detection of low-frequency mutations with CE has been reported by conducting STA with FFPE samples, detecting mutations ranging from 1 to 5%^7^. STA is a modality that extends primers with multiple bases to separate the base length between wild-type and mutant peaks on electropherograms, leading to relatively easy detection of mutant peaks with nearby and low signal intensity compared with SNaPshot^7^. Similarly, by conducting single base extension reactions with cell line-derived gDNA, mutations below 1% were detected by separating fluorescently labeled substrates into individual tubes for each colored labeling reaction^21^. However, detection of mutations below 1% with CE using clinical samples has not been reported.

The HiDy-CE addressed the challenge of signal saturation, enabling the identification of low-frequency mutations while preserving the distinguishability of wild-type signals, demonstrating the world’s first capability of detecting mutations less than 1% on CE using patient’s samples. Leveraging this HiDy technology, we successfully detected genetic mutations with very low VAF, ranging from 0.6 to 3.1%, in EUS-FNB samples obtained from patients with suspected tumors in the pancreaticoduodenal region. Despite variations in the VAFs among the genetic mutations, our data closely correlated with those obtained from dPCR. Notably, the HiDy-CE enabled the detection of low-frequency mutations. While it is possible to detect a single 1% mutant peak with the conventional-CE by carefully tuning sample concentration so that the signal intensity for both mutant and wild-type are within the detection range, this is difficult to achieve. It is almost impossible in the case of a multiplex assay where signal intensity varies.

Multiplexing is a potential advantage that CE technology originally possesses. When HiDy is combined with CE, multiplexing can be much more easily achieved because of its wider dynamic range. When implementing multiplexing, that is, measuring different types of genes simultaneously, multiple high-signal-intensity wild-type peaks are expected to appear in a single electrophoresis. Developing a reagent kit designed to ensure equivalent signal intensities of wild-type peaks across different genes on the conventional-CE is exceedingly challenging. In such cases, the HiDy-CE can simultaneously detect subtle signals from rare mutants of one gene and strong signals from abundant wild-type of other gene without saturation due to its wide dynamic range, whereas the conventional-CE suffers from signal saturation (Suppl Fig. 5).

Although we focused solely on *KRAS* Codon12 and 13 mutations, constructing a reagent kit where peaks appear for simultaneous detection of over 400 alleles can be achieved with the HiDy-CE. One of the notable advantages of a CE sequencer lies in its capability to analyze a wide range of base lengths, enabling accurate analysis ranging from 50 to 500 base pairs with a resolution of 1 base pair. By achieving such multiplexing capability, the HiDy-CE emerges as an optimal choice for cost-effective molecular screening in early diagnosis, boasting low cost, short TAT, and the ability to simultaneously detect multiple mutations in oncogenes and tumor suppressor genes.

In the STA assay on the HiDy-CE, the total TAT amounts to 210 min, including 100 min for PCR, 30 min for post-PCR purification, 35 min for STA, 10 min for post-STA purification, and 35 min for CE, according to TrimGen’s instruction. With modifications to the reagents, further optimization of PCR and STA conditions could potentially reduce the total TAT. In contrast, targeted amplicon sequencing takes 18 h to 1.5 days, and dPCR requires 160 min. The HiDy-CE not only has shorter TAT than targeted amplicon sequencing but is also comparable to dPCR. With further optimization, the HiDy-CE could achieve a TAT equivalent to dPCR. Although dPCR has a shorter TAT, by implementing the reagent kit described above, the HiDy-CE, with potential multiplexing capabilities could surpass both technologies in efficiency.

While our study demonstrated promising results, it is important to note several potential limitations. First, we recognize the possibility of the HiDy-CE detecting non-specific mutations (Suppl Table 1). These may arise due to the inherent characteristics of the analytical method or experimental conditions, potentially affecting its ability to discriminate specific mutations. Therefore, we are developing more specific detection methods and implementing strategies to exclude non-specific signals. Second, the limit of detection varied from 0.6 to 3.1% depending on the variant of the genetic mutation. Additional confirmation of these detection limits is warranted to ensure their reliability in actual clinical scenarios, necessitating a re-evaluation under different sample types and experimental conditions.

In conclusion, we developed an enhanced CE sequencer using a High Dynamic range fluorescence acquisition method (HiDy-CE). By minimizing the size of the hardware binning region and increasing the number of them, we have expanded the dynamic range, making this sequencer well-suited for STA-based mutation detections. Through further optimization of reagents and the development of multiplex detection systems for broader simultaneous analysis, the HiDy-CE is poised to provide a sensitive assay for detecting low-frequency mutations in minute tissue samples, opening doors for future clinical applications.

## Methods

### Patient samples

FFPE tissues were obtained from four patients with resectable pancreatic disease admitted to Asahikawa Medical University between 2018 and 2021. Residual tissues from EUS-FNB were obtained from 34 patients with pancreatic disease at Asahikawa Medical University between 2018 and 2023. The study protocol for patient-tissue collection and analysis was approved by the Asahikawa Medical University Research Ethics Committee (#21138). The study was conducted in accordance with the Declaration of Helsinki. Written informed consent was obtained from all patients before enrollment.

### EUS-FNB specimen collection

For histological diagnosis, EUS-FNB sampling was performed using a 22-gauge Franseen biopsy needle (Acquire; Boston Scientific, Marlborough, MA). Histologic cores obtained through multiple punctures, typically two to three, and collected in a Petri dish. The collected tissues were then fixed in FFPE, sectioned into 4- or 10-µm thick slices, and stained with H–E or used for gDNA isolation (Suppl Fig. 3).

### DNA preparation

Mutation profiling of patients’ samples from surgically resected tumor and normal pairs of FFPE specimens was previously analyzed using amplicon targeted sequencing. Tumor specimens were selected, each harboring *KRAS* mutations G12V, G12D, G12R, and G13D, and unstained Sects. (10-µm thick, 1–5 sections) were prepared and used for the isolation of gDNA^22^. Similarly, sections from EUS-FNB specimens (10-µm thick, 5 sections) were used for gDNA isolation. The purified gDNA was quantified using a Qubit dsDNA HS Assay Kit on a Qubit 4 Fluorometer (Thermo Fisher Scientific).

### Sample preparation

For the dilution series of gDNA extracted from cell lines, reference standards comprising 50% *KRAS* G12V, G12D, G12R, G13D, and wild-type gDNA were purchased from Horizon Discovery (Cambridge, UK). The mutation and wild-type gDNA were then combined to achieve the final VAFs from 0.1 to 5%. Similarly, for the dilution series of FFPE samples, gDNA extracted from tumor and normal tissues were used. Dilution series were prepared to yield final VAFs of 0.1 to 5% for subsequent analysis.

### Shifted termination assay

PCR amplification and STA reactions were conducted using the C1000 Touch™ Thermal Cycler (Bio-Rad Laboratories, Inc. Hercules, CA). Samples were analyzed using the *KRAS* Mutation Detection Kit (TrimGen, Sparks Glencoe, MD) following the manufacturer’s protocol. Briefly, DNA samples were diluted with Ambion Nuclease-free water (Thermo Fisher Scientific) to achieve a final concentration of 5 µg/µL. For each sample, a mixture comprising 18 µL of master mix, 1 µL of *KRAS* PCR primers and 2 µL of DNA sample was prepared. The PCR amplification protocol included an initial denaturation at 94 °C for 5 min, followed by 35 cycles of denaturation at 94 °C for 30 s, annealing at 52 °C for 30 s, extension at 72 °C for 30 s, and final extension at 72 °C for 5 min. Subsequently, 4 µL of PCR products were combined with 11 µL of clean-up enzymes and incubated at 37 °C for 25 min, followed by denaturation at 95 °C for 5 min.

Next, 2 µL of the clean-up sample was mixed with 11 µL of *KRAS* EM-12 or *KRAS* EM-13 and 2 µL of *KRAS* DP-12 or *KRAS* DP-13 for the STA reaction. The STA protocol included initial denaturation at 94 °C for 4 min followed by 20 cycles of denaturation at 94 °C for 20 s, annealing at 60 °C for 20 s, and extension at 70 °C for 20 s. The STA products were then cleaned using TF-50 filter tips. Finally, 3 µL of the solution was mixed with 15 µL of loading buffer and subjected to CE.

### HiDy-CE

Electrophoresis was conducted using either the compact CE sequencer DS3000 (Hitachi High-Tech, Tokyo, Japan) or the HiDy-CE. Both sequencers shared the same hardware configuration, featuring four 36-cm long capillaries and a CCD image sensor for fluorescence detection, with fluorescence projected onto the CCD image sensor covering a region of 3 × 240 pixels per capillary. However, the size of the region, hardware binning region, where the fluorescence was converted into analog-to-digital signals, differed between the two sequencers. In the DS3000, conversion occurred in units of 3 × 12 pixels, resulting in 20 hardware binning regions per capillary. In contrast, the HiDy-CE executed the conversion in units of 3 × 1 pixels, yielding 240 hardware binning regions per capillary. With the HiDy-CE, the 240 hardware binning regions were grouped in increments of 12, forming a total of 20 software binning regions (Fig. 1A).

Despite the same saturation level per hardware binning region between both sequencers, the total saturation level of the HiDy-CE increased due to the larger number of hardware binning regions. The HiDy-CE thus expanded the dynamic range by a factor of 8.09 by altering the signal output from the CCD image sensor. The signal output from the 20 hardware binning regions was de-convoluted into 4 dye signals to mitigate spectral overlap between the 4 dyes^23^.

### Electrophoresis

Electrophoresis analysis was conducted using the DS3000 and HiDy-CE. The electrophoresis conditions were as follows: an injection time of 9 s, run voltage of 12.5 kV, run time of 1280 s, and operating temperature of 55 °C. The injection of the sample into the capillary was conducted under the normal injection voltage of 1.6 kV, but experiments were also conducted at injection voltages of 0.5 kV (Suppl Fig. 1A) and 4.8 kV (Fig. 1B). POP-6 (Thermo Fisher Scientific) was used as the separating polymer.

To identify the peaks of interest, a control sample containing all alleles to be identified is electrophoresed in advance, prior to analyzing the target samples. The control sample enable precise determination each allele’s position. The maximum signal intensity within a range of ± 5 positions from the peak position in the control sample were recorded as the signal intensity for each allele.

Thresholds for the presence of mutations were set for each mutation type as the mean value plus three standard deviations at input VAF 0%. The input VAF at which all eight replicates exceeded the threshold was considered detection limits.

### Targeted amplicon sequencing and digital PCR

Targeted amplicon sequencing and dPCR were conducted using previously reported methods^22^. A custom DNA sequencing panel was used for mutation profiling of eight genes related to PDA. Additionally, 10 ng of DNA was amplified using this panel, and sequencing libraries were subsequently constructed.

## Supplementary Information

Supplementary Information 1.

Supplementary Information 2.

## Data Availability

The data supporting the findings of this study are available from the corresponding author upon reasonable request.
