# Supplementary material for: High dynamic range capillary electrophoresis method for sensitive detection of low-frequency driver mutations
Source: Sci Rep. 2025 Jul 1;15:21241. doi: 10.1038/s41598-025-01884-5 (PMC12218273; doi:10.1038/s41598-025-01884-5)
Supplement: Supplementary file 2 — Supplementary Information 2. [file 41598_2025_1884_MOESM2_ESM.pptx]

## Slide 1
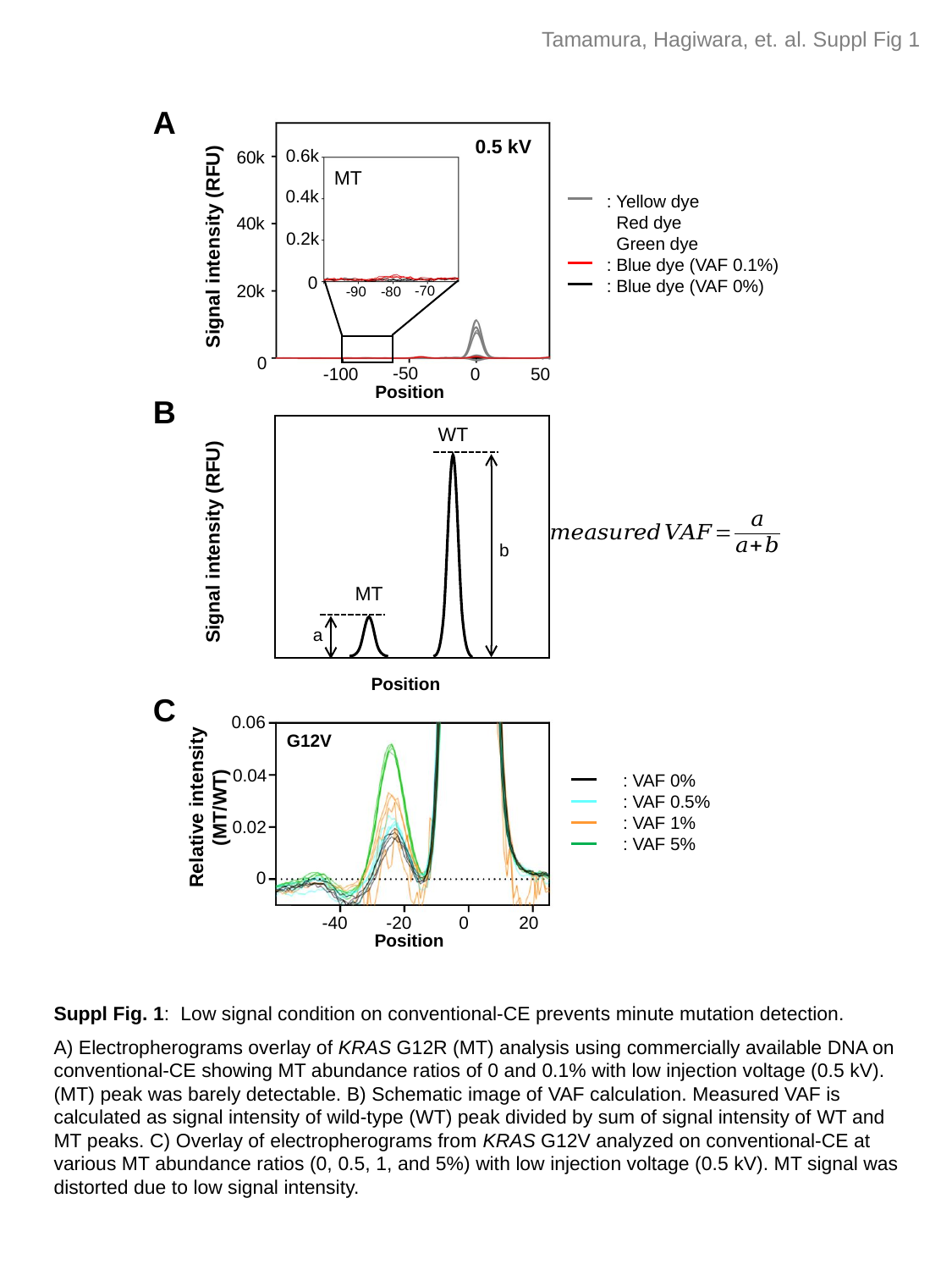

Tamamura, Hagiwara, et. al. Suppl Fig 1
A
0.5 kV
0.6k
60k
MT
0.4k
: Yellow dye
 Red dye
 Green dye
: Blue dye (VAF 0.1%)
: Blue dye (VAF 0%)
40k
0.2k
Signal intensity (RFU)
0
20k
-70
-90
-80
0
-50
0
50
-100
Position
B
WT
Signal intensity (RFU)
b
MT
a
Position
C
0.06
G12V
0.04
: VAF 0%
: VAF 0.5%
: VAF 1%
: VAF 5%
Relative intensity
(MT/WT)
0.02
0
-40
-20
0
20
Position
Suppl Fig. 1: Low signal condition on conventional-CE prevents minute mutation detection.
A) Electropherograms overlay of KRAS G12R (MT) analysis using commercially available DNA on conventional-CE showing MT abundance ratios of 0 and 0.1% with low injection voltage (0.5 kV). (MT) peak was barely detectable. B) Schematic image of VAF calculation. Measured VAF is calculated as signal intensity of wild-type (WT) peak divided by sum of signal intensity of WT and MT peaks. C) Overlay of electropherograms from KRAS G12V analyzed on conventional-CE at various MT abundance ratios (0, 0.5, 1, and 5%) with low injection voltage (0.5 kV). MT signal was distorted due to low signal intensity.

## Slide 2
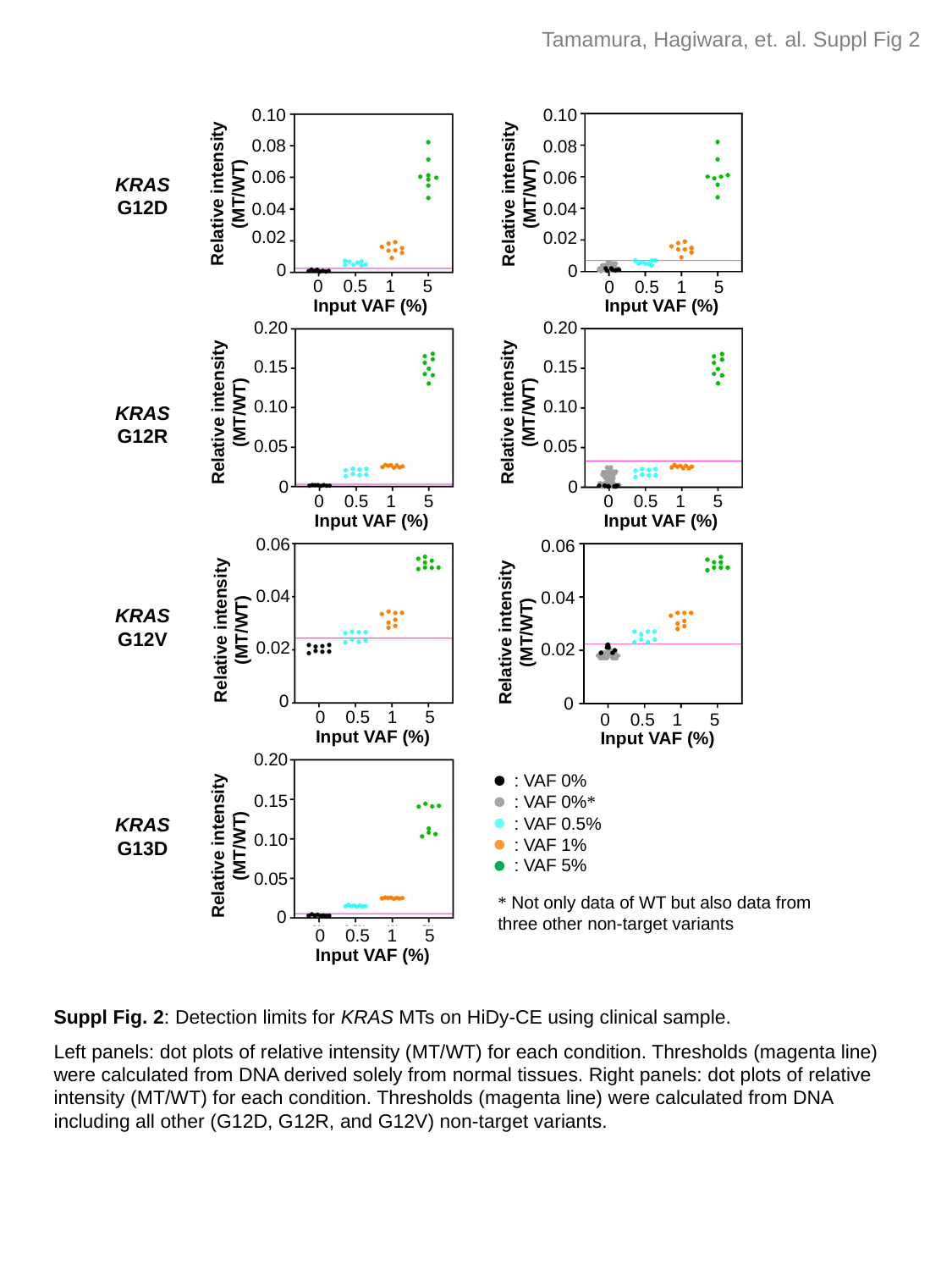

Tamamura, Hagiwara, et. al. Suppl Fig 2
0.10
0.10
0.08
0.08
0.06
0.06
KRAS
G12D
Relative intensity
(MT/WT)
Relative intensity
(MT/WT)
0.04
0.04
0.02
0.02
0
0
0
0.5
1
5
0
0.5
1
5
Input VAF (%)
Input VAF (%)
0.20
0.20
0.15
0.15
Relative intensity
(MT/WT)
Relative intensity
(MT/WT)
0.10
0.10
KRAS
G12R
0.05
0.05
0
0
0
0.5
1
5
0
0.5
1
5
Input VAF (%)
Input VAF (%)
0.06
0.06
0.04
0.04
KRAS
G12V
Relative intensity
(MT/WT)
Relative intensity
(MT/WT)
0.02
0.02
0
0
0
0.5
1
5
0
0.5
1
5
Input VAF (%)
Input VAF (%)
0.20
: VAF 0%
: VAF 0%*
: VAF 0.5%
: VAF 1%
: VAF 5%
0.15
KRAS
G13D
Relative intensity
(MT/WT)
0.10
0.05
* Not only data of WT but also data from three other non-target variants
0
0
0.5
1
5
Input VAF (%)
Suppl Fig. 2: Detection limits for KRAS MTs on HiDy-CE using clinical sample.
Left panels: dot plots of relative intensity (MT/WT) for each condition. Thresholds (magenta line) were calculated from DNA derived solely from normal tissues. Right panels: dot plots of relative intensity (MT/WT) for each condition. Thresholds (magenta line) were calculated from DNA including all other (G12D, G12R, and G12V) non-target variants.

## Slide 3
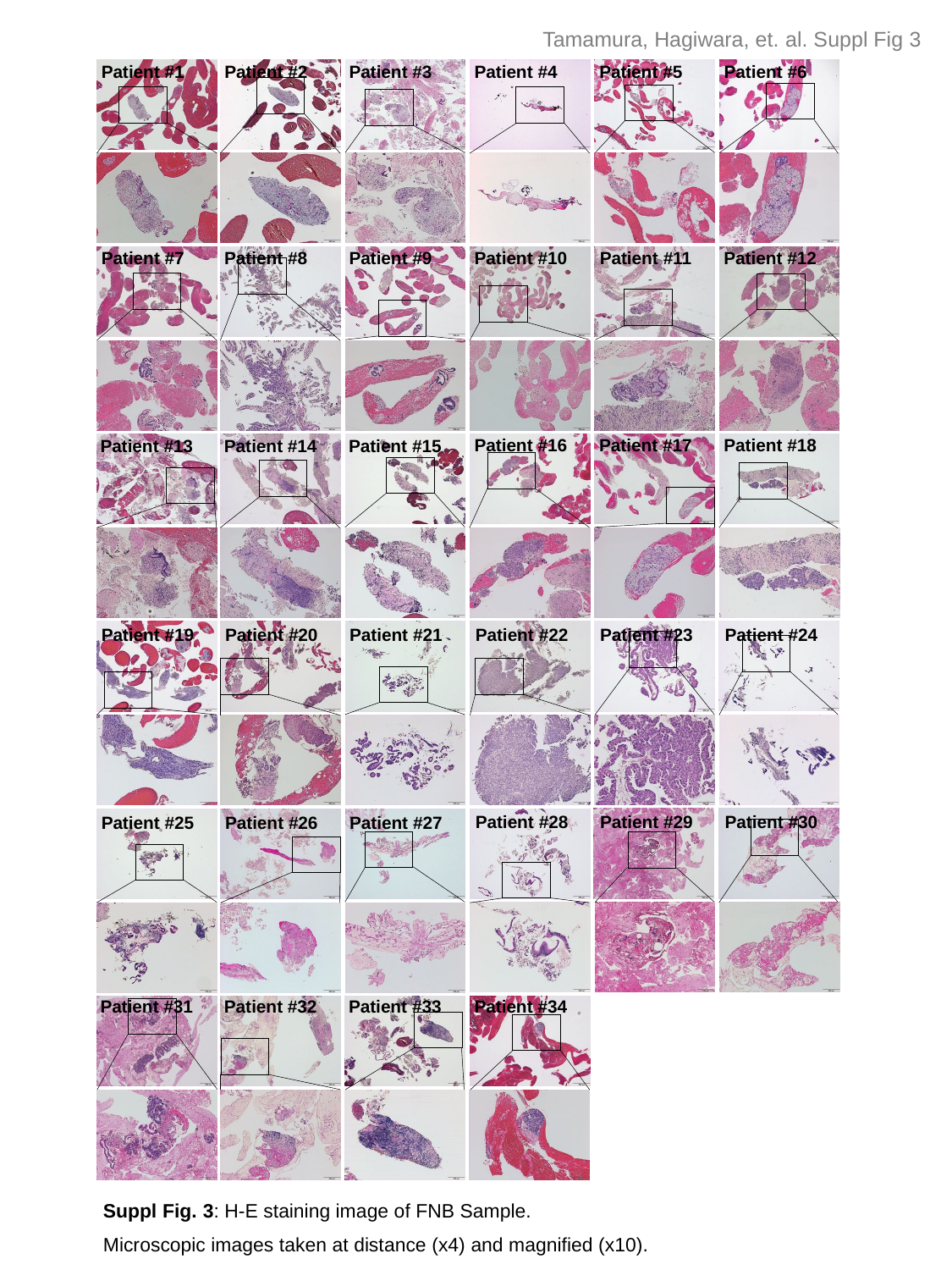

Tamamura, Hagiwara, et. al. Suppl Fig 3
Patient #1
Patient #2
Patient #3
Patient #4
Patient #5
Patient #6
Patient #10
Patient #11
Patient #12
Patient #7
Patient #8
Patient #9
Patient #16
Patient #17
Patient #18
Patient #13
Patient #14
Patient #15
Patient #22
Patient #23
Patient #24
Patient #19
Patient #20
Patient #21
Patient #28
Patient #29
Patient #30
Patient #25
Patient #26
Patient #27
Patient #34
Patient #31
Patient #32
Patient #33
Suppl Fig. 3: H-E staining image of FNB Sample.
Microscopic images taken at distance (x4) and magnified (x10).

## Slide 4
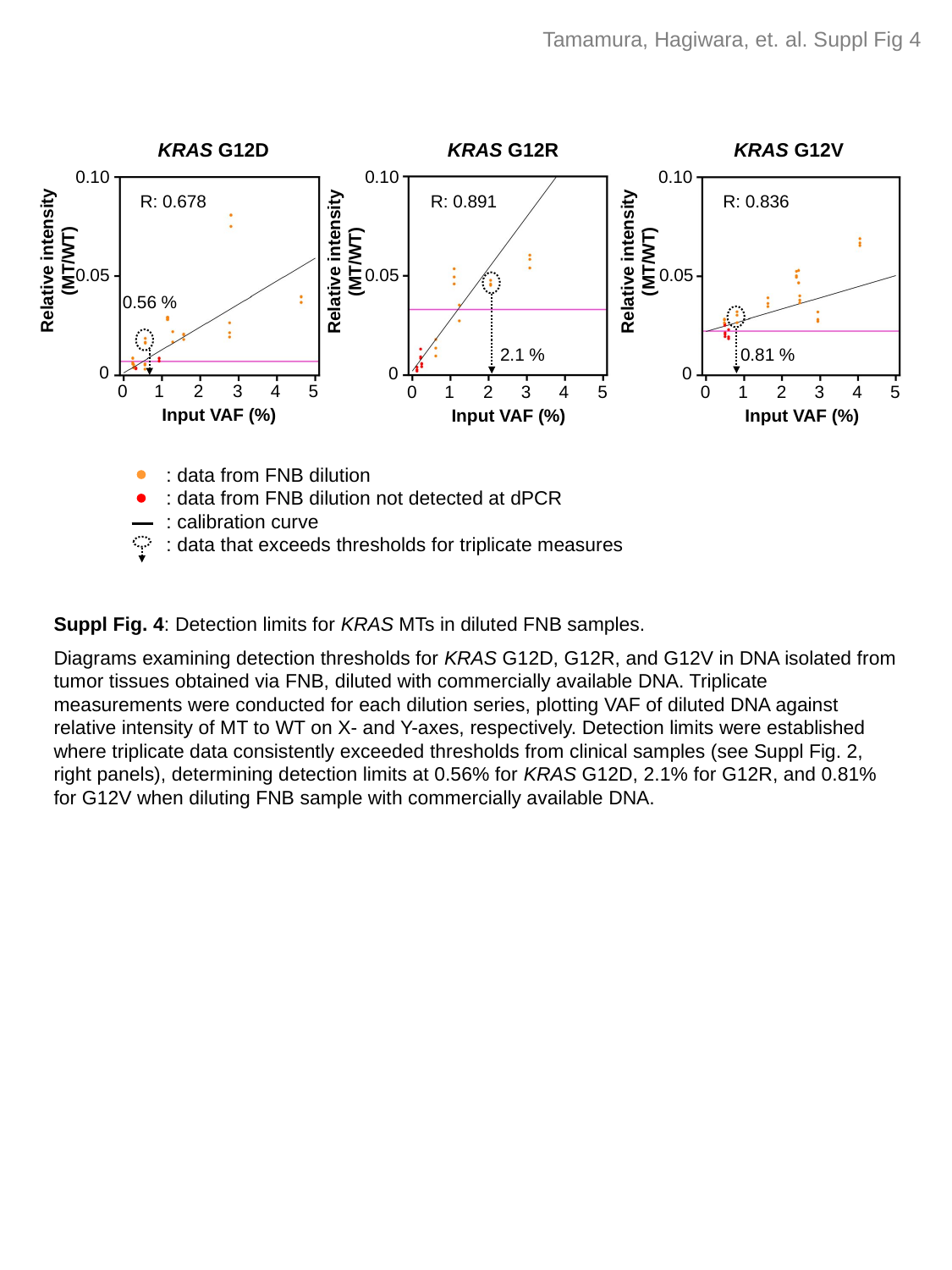

Tamamura, Hagiwara, et. al. Suppl Fig 4
KRAS G12D
KRAS G12R
KRAS G12V
0.10
0.10
0.10
R: 0.678
R: 0.891
R: 0.836
Relative intensity
(MT/WT)
Relative intensity
(MT/WT)
Relative intensity
(MT/WT)
0.05
0.05
0.05
0.56 %
0.81 %
2.1 %
0
0
0
0
1
2
3
4
5
0
1
2
3
4
5
0
1
2
3
4
5
Input VAF (%)
Input VAF (%)
Input VAF (%)
 : data from FNB dilution
 : data from FNB dilution not detected at dPCR
 : calibration curve
 : data that exceeds thresholds for triplicate measures
Suppl Fig. 4: Detection limits for KRAS MTs in diluted FNB samples.
Diagrams examining detection thresholds for KRAS G12D, G12R, and G12V in DNA isolated from tumor tissues obtained via FNB, diluted with commercially available DNA. Triplicate measurements were conducted for each dilution series, plotting VAF of diluted DNA against relative intensity of MT to WT on X- and Y-axes, respectively. Detection limits were established where triplicate data consistently exceeded thresholds from clinical samples (see Suppl Fig. 2, right panels), determining detection limits at 0.56% for KRAS G12D, 2.1% for G12R, and 0.81% for G12V when diluting FNB sample with commercially available DNA.

## Slide 5
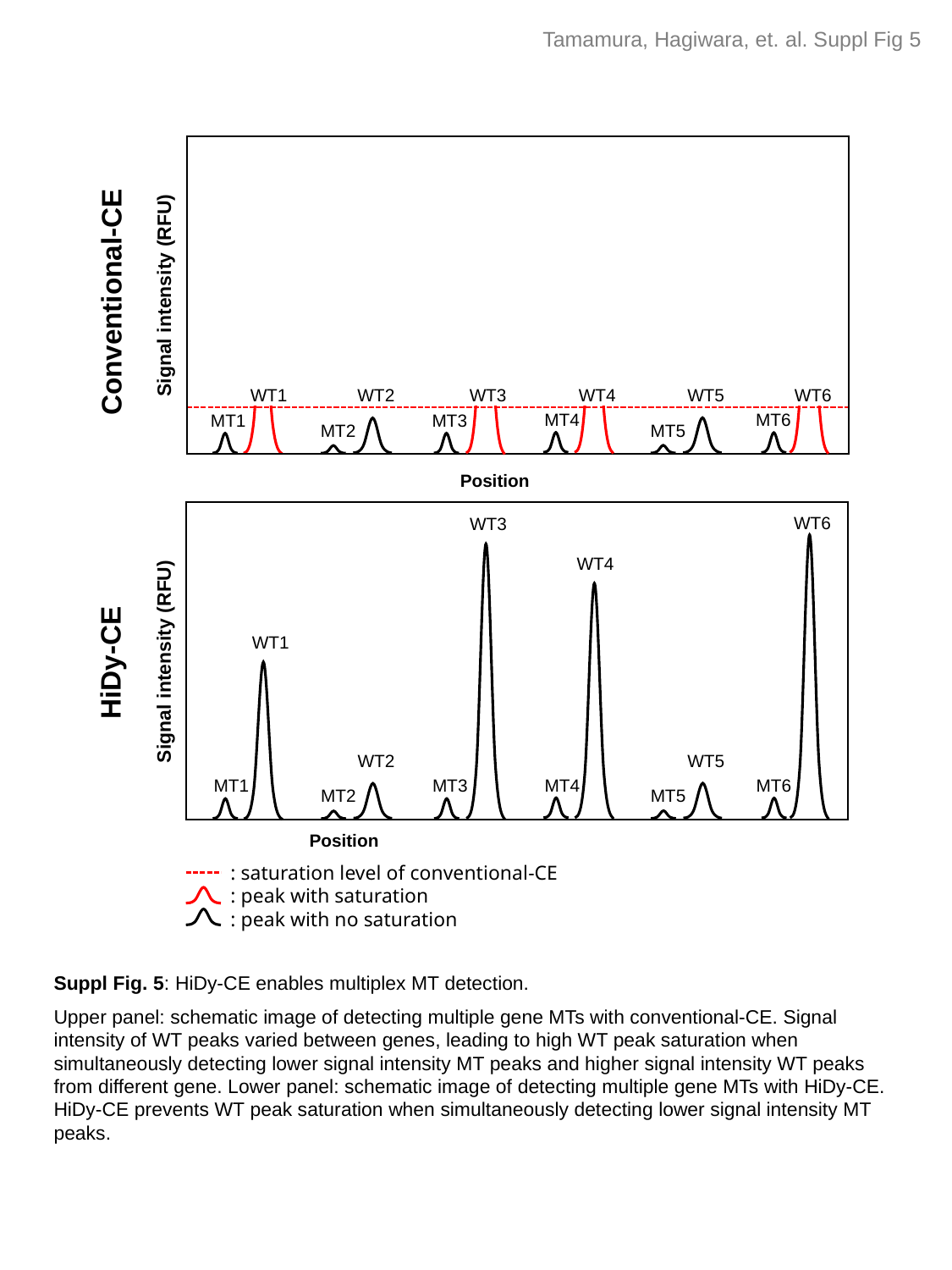

Tamamura, Hagiwara, et. al. Suppl Fig 5
Conventional-CE
Signal intensity (RFU)
WT1
WT2
WT3
WT4
WT5
WT6
MT4
MT6
MT1
MT3
MT5
MT2
Position
WT6
WT3
WT4
WT1
HiDy-CE
Signal intensity (RFU)
WT2
WT5
MT4
MT6
MT1
MT3
MT5
MT2
Position
: saturation level of conventional-CE
: peak with saturation
: peak with no saturation
Suppl Fig. 5: HiDy-CE enables multiplex MT detection.
Upper panel: schematic image of detecting multiple gene MTs with conventional-CE. Signal intensity of WT peaks varied between genes, leading to high WT peak saturation when simultaneously detecting lower signal intensity MT peaks and higher signal intensity WT peaks from different gene. Lower panel: schematic image of detecting multiple gene MTs with HiDy-CE. HiDy-CE prevents WT peak saturation when simultaneously detecting lower signal intensity MT peaks.
